# Cellular senescence induction by retinoid on adult T-cell leukemia cells

**DOI:** 10.1186/1742-4690-8-S1-A31

**Published:** 2011-06-06

**Authors:** Yasuhiro Maeda, Masaya Kawauchi, Chikara Hirase, Terufumi Yamaguchi, Jun-ichi Miyatake, Itaru Matsumura

**Affiliations:** 1 Department of Hematology, National Hospital Organization Osaka Minami Medical Center, Kawachinagano, Osaka, 586-8521, Japan; 2 Department of Hematology, Kinki University School of Medicine, Osaka-Sayama, Osaka, 589-8511, Japan

## Background

It has been reported that retinoid (all-trans retinoic acid: ATRA or tamibaroten: Am-80) inhibits growth in HTLV-1-positive T-cell lines and fresh cells from patients with adult T-cell leukemia (ATL) in vitro. In this study, we focused in role of cellular senescence in the treatment with retinoid.

## Methods

1. Cellular senescence was observed by senescence-associated β-galacsidase (SA β-Gal) staining.

2. Gene expression of cyclin dependent kinase, Tax was performed by RT-PCR.

3. To determine whether or not telomere attrition was occurred, TRAP assay was performed.

## Results

1. SA β-Gal positive cells were observed in spontaneous culture without retinoid on HTLV-I (+) T-cell lines (HUT102, MT-2, MT-4, ED40515 and ATL-2) or primary cells from acute ATL patients, but not on HTLV-I (-) T cell lines (Jurkat and MOLT-4). By treatment with retinoid, those number of SA β-Gal positive cells was increased significantly on HTLV-I (+) T-cell lines, but not on HTLV-I (-) T-cell lines (MOLT-4 and Jurkat).

2. Expression of CDK inhibitor, p16INK4a , was enhanced in all of HTLV-I (+) T-cell lines. 3. In TRAP assay, no inhibition of telomere activity was observed in retinoid treated HTLV-I (+) T-cell lines, indicative of premature cellular senescence.

## Conclusions

It has been reported that oncogene induced stress (OIS) induced cellular senescence. Tax gene may act as an oncogene in ATL cells, and retinoid facilitated the cellular senescence resulting in cell death. Taken together, retinoid therapy may be a reasonable therapy with cellular senescence induction in addition of OIS.

